# “We’re on a Merry-Go-Round”: Reflections of Patients and Carers after Completing Treatment for Sarcoma

**DOI:** 10.3390/curroncol28040263

**Published:** 2021-08-06

**Authors:** Rhys Weaver, Moira O’Connor, Richard Carey Smith, Dianne Sheppard, Georgia K. B. Halkett

**Affiliations:** 1 Curtin School of Nursing, Faculty of Health Sciences, Curtin University, Perth, WA 6845, Australia; rhys.weaver@curtin.edu.au; 2 WA Cancer Prevention Research Unit (WACPRU), School of Population Health, Discipline of Psychology, Faculty of Health Sciences, Curtin University, Perth, WA 6845, Australia; m.oconnor@curtin.edu.au; 3 Department of Orthopaedic Surgery, Sir Charles Gairdner Hospital, Perth, WA 6009, Australia; Richard.Careysmith@health.wa.gov.au; 4 Perth Children’s Hospital, Perth, WA 6009, Australia; 5 Perth Orthopaedic and Sports Medicine Centre, Perth, WA 6005, Australia; 6 Accident Research Centre/Office of the Provost and Senior Vice-President, Monash University, Clayton Campus, Melbourne, VIC 3800, Australia; dianne.sheppard@monash.edu

**Keywords:** sarcoma, survivorship, oncology, caregivers, qualitative research, rare cancer

## Abstract

Sarcoma is a rare cancer that has a significant impact on patients’ and carers’ quality of life. Despite this, there has been a paucity of research exploring the diverse experiences of patients and carers following sarcoma treatment. The aim of this study was to explore patients’ and carers’ reflections on life after treatment for sarcoma. A qualitative research design with a social constructionist epistemology was used. Participants included patients previously treated for sarcoma (*n* = 21) and family carers of patients treated for sarcoma (*n* = 16). Participants completed semi-structured interviews which were analysed using thematic analysis. Three primary themes were identified: “This journey is never going to be over”, “But what happens when I am better?”, and finding a silver lining. Participants represented sarcoma as having a long-term, and sometimes indefinite, threat on their life that they had limited control over. Conclusions: This study highlight the heterogeneous and ongoing needs of sarcoma survivors and their families. Patients and carers strove to translate their experiences in a meaningful way, such as by improving outcomes for other people affected by sarcoma. Parental carers in particular attempted to protect the patient from the ongoing stress of managing the disease.

## 1. Introduction

Sarcoma is a heterogeneous group of bone and soft-tissue cancers accounting for approximately 15% of paediatric cancers and 1% of all adult cancers in Australia [1,2]. There are prolonged intervals leading up to diagnosis, likely a product of the disease’s rarity and unfamiliarity [3,4]. Treatment often requires wide surgical resections or limb-salvage, alongside chemotherapy and/ or radiation-therapy [5]. Sarcoma patients and carers experience a range of unmet needs from diagnosis through to post-treatment, such as lack of psychosocial support, financial support, and patient-specific information [6]. Alongside the unmet needs, the effects from treatment and the malignancy can create long-term cosmetic and functional issues for patients and reduced quality of life [4].

There is a paucity of research exploring life after sarcoma treatment, despite the substantial impact diagnosis and treatment have on both patients and carers. Sarcoma patients have described finishing treatment as a traumatic experience, with the perception that significant and ongoing work lies ahead of them [7]. This ongoing work coincides with more infrequent contact with their treating specialists. Parsons and Eakin proposed that life after sarcoma treatment includes three types of work: illness work, identity work, and vocational work [7]. Illness work refers to work involved in managing their health, such as by regularly consulting health professionals. Identity work refers to the way patients to come to terms with changes in their identity, such as having a different appearance, reduced level of functionality, and different perspective on life. Vocational work is the recommencement of employment which can often be challenging for people affected by cancer [8].

Over and above the cancer survivor themselves, cancer affects the entire family. Family carers (“carers”) provide informal caregiving support to their family member diagnosed with cancer and may experience commensurate or greater distress than the patient [9]. Similar to patients, cancer carers must manage anxiety over a possible recurrence and manage a changed life after treatment [10]. Björk et al. considered the different perspectives on life held by parent carers relative to their children; parent carers may more accurately view the end of treatment as a partial recovery and the beginning of a period of uncertainty [11]. Finishing treatment may also provide sarcoma carers with the opportunity to consider their own needs and reflect on how their life has been affected by sarcoma [12].

Life after sarcoma treatment is complicated by the lasting physical and psychosocial impacts of the disease [13,14]. This is exacerbated by the somewhat naïve and commonly perceived expectation that patients and carers simply resume life as before treatment, despite this being rarely possible [15]. Even though post-treatment issues in cancer survivorship have been widely reported, recent reviews suggest that patients are still feeling unprepared and inadequately supported after treatment [14,16]. Sarcoma patients have described needing improved access to physiological and psychological support [17]. Patients who do not receive adequate support may be less able to adjust to physical impairments and may have poorer psychosocial wellbeing [17]. 

### Aim

Research exploring life after sarcoma treatment has been limited, and of that research there has been a focus on “return” to work. Framing life after treatment in this way can be misleading as patients and carers rarely “return” to where they left off; they often have different goals, perspectives, and relationships than before treatment [7]. There has also been a focus on measuring quality of life and functional outcomes, rather than exploring the diverse and complex experiences of patients and carers [18]. The aim of this study was to fill that void by examining the way patients and carers reflected on life after treatment for sarcoma.

## 2. Materials and Methods

This study used an exploratory qualitative research design with a social constructionist interpretive framework. Social constructionism is the epistemology which assumes that individuals have unique experiences through which they construct meaning [19]. The researchers co-constructed meaning by asking questions to direct the conversation of the interview and through the coding process.

### 2.1. Theoretical Framework

Leventhal’s Common Sense Model (CSM) of self-regulation informed this study [20]. The CSM suggests that when individuals experience an emotional, physical, or social risk, the way they represent this risk informs how they choose to manage it [21]. An illness representation is the way a health threat is labelled, and beliefs about the cause, consequence, duration, and level of control over the threat. How the threat is represented then informs the way the individual manages the threat, such as by consulting a health professional. This model is dynamic in that the health threat, illness representation, and self-management inform each other may change over time. The CSM of self-regulation was chosen as previous research has found that patients actively manage a variety of threats to their physical and mental health after treatment. The model facilitated data analysis by providing a framework to understand the relationship between participants’ concerns and the way they manage their concerns.

### 2.2. Participants

Participants were eligible if they could converse in English and were over the age of 16 at the time of the interview. Patients were required to have been diagnosed with sarcoma in the last 15 years and completed treatment for sarcoma. Carers were required to have cared for a friend or relative who had been diagnosed with sarcoma in the last 15 years, including patients under the age of 16. Eligible patients were identified by health professionals working in a tertiary hospital in Perth, Western Australia (WA), Australia. Word-of-mouth was used to recruit additional patients and carers through the “Sock it to Sarcoma! Foundation”. Participating patients were invited to recruit their family carers and vice versa. Participants were not required to join as a dyad. Most patients and carers were interviewed separately; however, four pairs of patients and carers were interviewed together at their request. This was to allow potentially distressed participants to have their loved one available as support during the interview.

### 2.3. Data Collection

All participants provided informed consent before being interviewed. The interviewer informed participants of their occupation, place of occupation, and credentials prior to the interview. Interviews were facilitated with a semi-structured interview guide containing questions about the participant’s experience with sarcoma and life after treatment. The interview guide was reviewed by patient and carer consumers prior to commencement and has been previously published online [22]. Interviews were conducted by RW, GH, and MOC who each had experience conducting qualitative research interviews. Interviews were conducted either at a mutually convenient location (e.g., their home) or over the phone. All interviews were audio-recorded and transcribed verbatim. Participants were interviewed between 25th June 2018 and 2nd May 2019.

### 2.4. Data Analysis

Interview transcripts were analysed inductively using thematic analysis. NVivo 12 was used to manage the data. Thematic analysis involved familiarisation with data, generating initial codes, developing themes, defining and naming themes, and producing the write-up. Initial codes were generating by identifying and labelling data relevant to the research question. Themes were formed by grouping similar codes together to represent patterns in the data. Patient and carer data were analysed together as a single sample., however, the researchers did not assume that each group would have the same experiences and were mindful of potential differences across the participants.

### 2.5. Data Saturation

Data saturation is said to occur when interviews do not provide any new relevant information [23]. Interviews were conducted concurrently with data analysis after the first interview was completed. When an interview generated no new codes, an additional interview was conducted to verify that data saturation had been reached. Recruitment ended when each group had reached data saturation. We were aware that data saturation is a contested concept and focused on collecting rich data during interviews and only closing recruitment when no new relevant information was obtained.

### 2.6. Rigour

During the research process we were mindful of how our expectations and biases may influence the research process, particularly during data collection and analysis. Reflexive journaling was used throughout the research process to identify and manage our potential biases. Coding was done by RW and GH, both of whom had experience with thematic analysis. RW and GH conceptualised and developed the themes, which were reviewed by the wider authorship team. The study has been reported using the consolidated criterion for reporting qualitative research checklist (COREQ) [24].

### 2.7. Ethical Considerations

Ethical approval was granted from the university (HRE2018-0246) and participating tertiary hospital (EC00271). This research is part of a larger project exploring the patient care of sarcoma in WA. We have previously published on delays in diagnosis and the unmet needs of patients and carers elsewhere [4,6,12]. Multiple studies were used to provide thorough consideration of different aspects of patient and carer care, which could not be captured in one publication alone.

## 3. Results

Twenty-one patients and 16 carers were interviewed. Most participants were interviewed face-to-face (*n* = 25), with the remainder participating in phone interviews (*n* = 12). Mean interview time for patients was 53 min (SD = 21 min) and mean interview time for carers was 52 min (SD = 20 min). Patient and carer demographics are reported in Table 1 and Table 2, respectively.

Three overarching themes were formed based on the ways in which participants conceptualised sarcoma since finishing treatment. The themes are: “This journey is never going to be over” which includes the sub-theme of surveillance, “But what happens when I am better?”, and finding a silver lining. Exemplar quotes are supported with the patient’s sex or the carer’s relationship with the patient and the time since diagnosis (intervals represented as ≤2, 2–5, or ≥5 years post-diagnosis to de-identify data).

### 3.1. “This Journey Is Never Going to Be Over”

Participants characterised sarcoma as having an enduring impact on their life. Some patient participants had an expectation that “everything would just fall into place” after treatment (P12, male, ≤2 years post-diagnosis). However, ongoing medical and psycho-social concerns disrupted their desire to “get on with their life” (P01, female, 2–5 years post-diagnosis). For example, C05 suggested that the potential for heart failure created a perpetual threat against the patient’s health and their experience with sarcoma was not over after more than five years post-diagnosis:

*“This journey is never actually going to be over. We have to have constant heart checks because the chemo can affect your heart—in his 20s and 30s he may have to have a heart transplant. People who have no idea think that because he is finished treatment, he’s fine.” (C05, mother, ≥5 years post-diagnosis).*

Participants indicated that sarcoma had substantially disrupted their life, contributing to a loss of direction and control over their life. P24 explained that they had been so focused on managing the disease that they did not know what to do after completing treatment, particularly as they had to give up work:

*“People keep saying, ‘You’ve got to focus on your health and you’ve got to get better’, but at the moment it’s like, ‘Yeah, but what happens when I am better? I’m floundering.’ And I’ve never been without work.” (P24, female, ≤2 years post-diagnosis).*

Sarcoma appeared to have a long-term impact on participants who reported experiencing traumatic stress several years after diagnosis. One of the ways this stress affected both the carer and patient participants was their preparedness to recommence employment. For example, C06 and P03 highlighted the tension between feeling expected and financially pressured to start working and not being ready to go back:

*“I was starting to think about re-entering work and I didn’t get there, which is hard because I’m now three years past [patient’s] diagnosis, but it has taken three years to sort of get my head to a reasonable level of sanity. It has been really difficult. Post-traumatic stress is actually really common with kid’s cancer.” (C06, mother, 2–5 years post-diagnosis).*

*“I was getting stressed and my partner could see some anxiety happening there and I said, ‘I’ve got to go to work next week. I’ve got to be back in work.’ And she’s gone, ‘You’re in no state to go back to work.’ It was good to have that reaffirmation from her because my medical certificate’s running out. I’ve got to be back at work, and I didn’t feel ready to be back at work.” (P03, male, ≤2 years post-diagnosis).*

Patients and carers appeared to carry the stress of sarcoma in different ways. This was particularly apparent when parent carers were still stressed over the disease while the patient was more ambivalent: “I don’t think about it much anymore. Mum tends to be the worrier and I keep going on without it” (P09, male, 2–5 years post-diagnosis). A parent carer discussed that they managed the medical aspects of recovery to help the patient get on with their life:

*“[Patient] wanted to get back to school, wanted to get back into the sports team… [The parents] get the documents, find out when the next check is, work out when we had to go in and have the PET scan.” (C10, father, 2–5 years post-diagnosis).*

C06 explained that their child’s desire to distance themselves from sarcoma was to step away from the label and experience of being a cancer patient: “He doesn’t want it to define him. He doesn’t want to be the cancer kid.” (C06, 2–5 years post-diagnosis).

#### Surveillance

Surveillance is a subtheme of “this journey is never actually going to be over”, as patients needed regular scans and follow-up appointments for years after treatment. This sub-theme is a form of illness work and describes how participants managed the disease. Participants had a complex relationship with surveillance and had mixed emotional responses. For example, P02 did not like thinking about surveillance but recognised that it was a necessary part of managing the disease:

*“I don’t like to think about all the medical stuff that comes along with [surveillance].” (P02, female, 2–5 years post-diagnosis).*

*“But you’ve got to go and do it because you need that reassurance as time goes by.” (C02, mother, 2–5 years post-diagnosis).*

While participants acknowledged the chance of a recurrence was decreasing with each scan, the participants’ anxiety sometimes increased over time as the scans become further apart:

*“It’s a little bit scary for [patient] because she’s constantly had that reassurance of having a scan and knowing that it’s all good, and going through the 12 months the first time was really hard, not knowing that there was nothing going wrong.” (C04, mother, ≥5 years post-diagnosis).*

This ongoing cycle of anxiety and reassurance throughout surveillance was described as a “merry-go-round”, where patients and carers “felt good for another three months until it comes around again” (C16, husband, ≤2 years post-diagnosis). Not every patient was as stressed about surveillance however, as some felt there was no purpose in worrying about future recurrences and accepted that it was out of their control. These participants considered this to be a realistic representation of the nature of the disease:

*“You’ve got no point worrying about stuff that may never happen. You could walk out of here and get hit by a bus, but I’m not going to worry about that either. Just get on with it, get scanned every four months or whatever it is.” (P14, male, ≤2 years post-diagnosis).*

### 3.2. “But What Happens When I Am Better?”

This theme refers to the ways in which participants adjusted to the changes brought on by sarcoma, some of which may persist indefinitely. Sarcoma impacted the participant’s functionality, appearance, and lifestyle, and they emphasised a desire to “continue a normal life” after treatment (P04, male, 18, 3 years 8 months post-diagnosis). When discussing their loss of functionality, patients used normality to refer to their capacity before treatment and record their progress after treatment: “I’m absolutely amazed at how strong the knee came back. It’s pretty much back to normal” (P06, male, ≥5 years post-diagnosis).

It was evident that many participants were unable to operate as they could before treatment. Some participants recognised that they could still experience some level of normality, but would not be able to do everything that they could do before treatment. For example, P03 aimed to do the same activities as before treatment but at a more manageable level:

*“My attitude was to continue to do what I was doing before, albeit a little bit different and a lot slower. Well I do everything normal now, I’m back to driving a car. The thing I can’t do now is I can’t run on hard ground; but I can jog.” (P03, male, ≤2 years post-diagnosis).*

Some participants had to deal with a changed appearance after treatment, for example, C06 observed that after her son had surgery on his leg, “it took a lot of work to get [patient] to wear shorts, to feel okay about it” (C06, mother, 2–5 years post-diagnosis). Coming to terms with a new appearance was challenging as patients had to contend with what they previously looked like alongside social expectations of what they should look like. For example, P24 framed the difficulties of her changed appearance from the perspective of being a woman and wanting to look presentable for her daughter’s wedding:

*“When my hair fell out, that was the biggest thing. It’s not just your hair it’s your eyelashes, it’s your eyebrows—and then I had my daughter’s wedding that put more pressure on me because I had no eyelashes. Just things like that as a woman, that’s been the toughest, and how my body shape has changed. I’ve put on six kilos in the 12 months because I haven’t been able to exercise enough.” (P24, female, ≤2 years post-diagnosis).*

Participants appeared to become more comfortable with these changes over time, with P13 reflecting that “with more time, I’m more accepting of it…. this is me post-cancer”. However, they acknowledged that they were not prepared for how difficult post-treatment would be, particularly because finishing treatment had been characterised as the end of her experience with sarcoma:

*“I don’t think there’s enough discussion and awareness about all the stuff after treatment that you have to be prepared for and deal with. You finish treatment and it’s kinda like, ‘see ya, good luck, go live your life now’. And I think it has actually been harder after treatment than in treatment no one’s telling you what you have to do anymore, you’ve ‘won’ as they tend to say.” (P13, female, 2–5 years post-diagnosis).*

### 3.3. Finding a Silver Lining

This theme is about the ways participants found meaning from their experience with sarcoma. Several participants thought that while they were unlucky to be diagnosed with sarcoma, they were lucky to be in remission and to still be alive: “I got the unlucky card but at the same time I also go the Lotto (lottery) card” (P16, male, 2–5 years post-diagnosis). In contrast, some participants struggled to come to terms with survivorship when they knew other patients who had died: “How do I sit and tell my tale of woe to someone whose kids are dead? Why aren’t I dead? Why is her kid dead and I’m alive?” (P19, female, ≥5 years post-diagnosis).

Participants wanted to highlight how their experience with sarcoma had changed their life for the better, such as by bringing their family closer together:

*“I think we’re closer. We’re much more friendly and that might just be a result of us both being older, but it also just could be because we spent more time one on one that we probably would have.” (C08, brother, 2–5 years post-diagnosis).*

Generally, participants suggested that it was important to incorporate their experience with sarcoma into their life in a meaningful way. For example, P02 made an active decision to “look on the bright side of things, because there was no point wallowing in the negative side of life”. This participant reflected that their experience with sarcoma inspired their current studies and career aspirations to improve outcomes for those affected by sarcoma:

*“I’ve got a real passion for medical science and my end goal is to work in cancer sarcoma research. I just want to find a solution to help others suffering from this pretty horrendous cancer.” (P02, female, 2–5 years post-diagnosis).*

P14 explained that surviving sarcoma gave them a “peek in the window” of what it’s like for patients, and drove them to do “whatever I can do to try and help the situation” (P14, male, ≤2 years post-diagnosis). Participants engaged in a variety of activities, such as fundraising, raising awareness about sarcoma, and offering their services:

*“I’ve done the HBF run. I’m going to do the Ride ‘n Stride. I’ve done the West Australian Sarcoma Awareness. Last year I was at [the hospital], shaking a tin, going around the hospital, and this year I was just on their display on their walkway.” (C01, wife, ≤2 years).*

C05 explained that contributing to a cause had a dual purpose as it provided an outlet for them to manage their own stress surrounding the disease while also helping other people:

*“It gave me a focus. I remember saying, ‘I can’t fix him, I can’t make this go away. I can only be there for him’. But I also need to keep my sanity and to feel like I’m doing something to make a difference and to help where I can.” (C05, mother, ≥5 years).*

## 4. Discussion

This study aimed to explore the experiences of patients and carers after completing treatment for sarcoma. The richness of the interview data provided a detailed account of the participants’ varied and complex experiences. Patients and carers detailed a range of health threats, including fears of recurrence, health consequences from the disease and treatment, and changes to their identity. Sarcoma was characterised by participants as an ongoing and enduring threat that they had limited control over. Participants had heterogeneous experiences with sarcoma and showed varying concerns, ways of adjusting, and representations of their health. While the issues raised by participants are not unique to sarcoma, the high rate of long term and chronic complications in sarcoma likely complicated their ability to adjust after finishing treatment [25].

Consistent with previous literature, patients had to adjust to changes in their life after finishing treatment. This process has been referred to as finding a “new normal”, “identity work”, or returning to a changed life [7,11,26]. A patient’s mental health can be significantly impacted if they are unable to step back into their life roles after treatment. An evaluation on quality of life in soft tissue sarcoma patients found that restricted participation in life roles and situations had the greatest negative affect on quality of life [27]. The current findings reflect previous research findings that adjusting to life after treatment is an active and ongoing process [7]. Reaching a point of active acceptance helped participants come to terms with their new identity, particularly cosmetic changes (such as scarring) which were outside of their control. Setting and achieving reasonable goals for their functionality also helped participants improve their mobility and feel a greater sense of normality.

Carers also experienced disruptions to their life roles, however they did not discuss how sarcoma had changed their lifestyle or identity to the same extent as patients. Carers can be so focused on the patient’s wellbeing that they do not recognise their own needs [12]. Some carers were unwilling to discuss their wellbeing even when they admitted to feeling more distressed about the disease than their loved one. Parent carers in particular took on a role of disease management to shield their child from the stress of the disease. For example, some parent carers managed all the patient’s appointments so the patient could focus on their schoolwork and social life. Previous research has also found that parents may sacrifice their own wellbeing to protect their children from negative psychosocial responses and trauma [10,28]. It is important, however, for carers to recognise that this should not come at the cost of their own wellbeing.

Many of the participants’ concerns were perceived to be in the future and outside their control, such as a recurrence or medical issues. An individual’s illness representation is closely linked to the degree of fear experienced [29] and greater fears of recurrence occur when cancer is viewed as a chronic and uncontrollable illness [30]. Cancer patients in general have a lower internal locus of control and loss of control, which can lead to decreased quality of life and poorer mental health outcomes [31,32]. Some participants (carers as well as patients) in this study found it distressing to not have control over the disease, while others readily accepted and were comfortable with it. This reinforces the heterogeneity of the experiences of sarcoma patients and the need to deliver individualised psychosocial assessment and support [33].

Participants, including carers, also looked for meaning in their experience with sarcoma in various ways. The main way that participants found meaning was by using their experience to improve outcomes for other individuals affected by sarcoma. Previous findings reported by Appau and Churchill found that individuals are more likely to volunteer and experience greater wellbeing when engaging with an organisation that they feel closer to, likely contributing to the participants’ positive experience of volunteering [34].

### 4.1. Implications

While the prevalence of post-treatment issues in sarcoma are widely reported, patients felt unprepared and inadequately supported for the challenges after treatment [7,35]. Participants were surprised at some of the issues they faced after finishing treatment, suggesting that there is scope to prepare patients and their families better for end of treatment. Given many aspects of sarcoma survivorship are outside the individual’s control, acceptance and commitment therapy (ACT) could be trialled with patients and carers to see if it improves outcomes post-treatment. Acceptance and commitment therapy (ACT) is particularly beneficial for issues outside of the individual’s control where the stressor is not easily changed or removed [36]. Encouraging patients to set achievable goals may also provide them with a sense of achievement and progress in areas that are in their control.

Providing regular and thorough screening post-treatment may assist health professionals in giving individualised referrals based on each patient’s needs. For example, the University of Michigan has a sarcoma survivorship clinic to provide long-term care by diagnosing and managing future complications in a timely manner [37]. This involves regular screening, such as for cardiac disease, skeletal-muscular dysfunction, depression, and anxiety; survivors are then provided with specialist referrals as needed. Similar services could be implemented by sarcoma specialist teams to provide greater emphasis on survivorship care. Survivors and carers should also be provided with vocational support to assist in their transition back into work.

### 4.2. Limitations

This study provides insight into some of the issues experienced by sarcoma patients and carers post-treatment. Further longitudinal research could provide an understanding of how patients and carers adapt to life after treatment over time. Longitudinal research may also ameliorate recall difficulties that participants may have had. The participants’ experiences may have been shaped by their membership with the “Sock it to Sarcoma!” Foundation, such as their strong desire to help others affected by sarcoma. The transferability of the study is also limited by our small sample size from a single country. Having a larger sample size and stratifying participants (e.g., by histology or treatment type) may have further highlighted unique aspects of sarcoma survivorship. Recent quality of life studies with large participant pools, such as PROSa (*n* = 1113) and SURVSARC (*n* = 1099), have observed differences in quality of life between sarcoma patients, such as by tumour location [38,39]. Although we aimed to achieve saturation with a diverse group of participants, there would be patients and carers we did not interview who are adjusting to life in different ways.

## 5. Conclusions

The current findings highlight the long-term consequences of sarcoma, including disrupted life roles, ongoing medical complications, and having to make sense of their illness. Participants represented sarcoma as a long-term threat that they had limited control over and had different ways of coping. Carers experienced similar psychosocial challenges as the patient, with some even taking on more responsibilities post-treatment to shield the patient from stress. Health professionals who work with patients and carers affected by sarcoma should be preparing them for life after treatment and providing individualised and holistic care.

## Figures and Tables

**Table 1:** Patient demographics (*n* = 21).

Characteristic	Number
*Age (years)* (Missing = 2)	
Mean	42 (SD = 18.21, Min = 15, Max = 78)
*Sex*	
Male	9
Female	12
*Histology* (Missing = 1)	
Osteosarcoma	4
Undifferentiated pleomorphic	3
Ewings	2
Chondrosarcoma	2
Synovial	2
Epithelioid	1
Rhabdomyosarcoma	1
Chordoma	1
Fibroblastic	1
Leiomyosarcoma	1
Liposarcoma	1
Malignant peripheral nerve sheath	1
*Tumour location*	
Head and neck	2
Lower extremities	11
Upper extremities	3
Pelvis	3
Spine	1
Torso	1
*Type*	
Bone	7
Soft tissue	14
Both	1
*Surgery*	
Resection	13
Amputation	4
Limb salvage	3
Bone excision	1
*Additional treatment*	
Chemotherapy	10
Radiation therapy	10
Targeted therapy	2
Hormonal therapy	1

SD = Standard Deviation. Min = Minimum. Max = Maximum.

**Table 2:** Carer demographics (*n* = 16).

Characteristic	Number
*Age (years)* (Missing = 2)	
Mean	51 (SD = 11.34, Min = 22, Max = 66)
*Sex*	
Male	4
Female	12
*Duration as a carer (months)* (Missing = 3)	
Mean (range)	34 (SD = 26.15, Min = 2, Max = 96)
*Relationship to patient*	
Mother	9
Father	1
Wife	3
Husband	2
Brother	1
*Age of patient at diagnosis (years)*	
Mean	26 (SD = 20.60, Min = 2, Max 62)
*Histology* (Missing = 2)	
Osteosarcoma	5
Ewings	4
Chondrosarcoma	1
Chordoma	1
Epithelioid	1
Rhabdomyosarcoma	1
Synovial	1
*Tumour location*	
Lower extremities	10
Head and neck	2
Upper extremities	2
Pelvis	1
Torso	1
*Type*	
Bone	9
Soft tissue	6
Both	1

SD = Standard Deviation. Min = Minimum. Max = Maximum.

## Data Availability

The data supporting the findings are available from the corresponding author upon reasonable request. The data is not publicly available due to privacy and ethical restrictions.
